# How Affective Relationships and Classroom Norms Shape Perceptions of Aggressor, Victim, and Defender Roles

**DOI:** 10.1002/ab.70020

**Published:** 2025-01-28

**Authors:** Tomáš Lintner, Adam Klocek, Ivan Ropovik, Lenka Kollerová

**Affiliations:** ^1^ Department of Educational Sciences Masaryk University Brno Czech Republic; ^2^ Institute of Psychology, Czech Academy of Sciences Brno Czech Republic; ^3^ Institute for Research and Development of Education Charles University Prague Czech Republic; ^4^ Faculty of Education University of Prešov Prešov Slovakia

**Keywords:** bullying, classroom bullying norms, defending, liking and disliking, multiplex social networks, physical aggression, SAOM, victimization

## Abstract

Reputational peer nominations are a common method for measuring involvement in aggression‐related behaviors, encompassing the roles of aggressor, victim, and defender, but may be influenced by students' affective (dis)liking relationships. This social network study investigated whether dyad‐ and group‐level (dis)liking relationships affect perceptions of classmates' involvement in physical aggression and explored the moderating roles of classroom moral disengagement and defending norms. The study employed a longitudinal design with two time points 6 months apart, encompassing 27 classrooms and 632 early adolescents. Using multiplex stochastic actor‐oriented modeling, we found that liking, but not disliking, significantly influenced perceptions. Liking a classmate increased the likelihood of perceiving them as a defender. Moreover, students' own perceptions (aggressor, victim, and defender nominations) were shaped by the perceptions of classmates they liked, while classroom moral disengagement reduced this influence for defender nominations. Results on classroom defending norms were mixed. Our findings underscore the importance of accounting for students' liking relationships and classroom‐level norms to reduce bias in peer nominations and improve the accuracy of assessments of aggression‐related behaviors.

## 1 Introduction

Physical aggression among peers is a pervasive phenomenon in schools (Kuppens et al. 2013; Tso et al. 2018) and meta‐analyzes provide robust evidence of positive associations between victimization–including victimization by physical aggression–and both internalizing and externalizing problems (Jagasia et al. 2024). Considering the universal presence of physical aggression in schools and its negative consequences, it is crucial to have accurate methods for identifying students' involvement in it. Typically, physical aggression in schools is studied within the bullying framework, because bullying presents particularly severe aggressive behaviors characterized by asymmetry of power and often also by repetitiveness (Solberg, Olweus, and Endresen 2007). Bullying, victimization, and defending by physical, verbal, social, or cyber aggressive acts can be identified by self‐reports or by two peer nomination methods–a direct relational approach or a reputational peer nominations approach (Hunter, Noret, and Boyle 2021). In the relational approach, students are asked to identify who attacks or defends them or other classmates (e.g., Huitsing and Monks 2018). This line of research demonstrated that development of physical aggression is interconnected with development of liking, with robust support for selection effects (Dijkstra and Berger 2018; Dijkstra, Berger, and Lindenberg 2011; Zhang, Liu, and Zhang 2020) and some support also for influence effects (Zhang, Liu, and Zhang 2020). In the reputational approach, students are asked to rate each other on specific items aiming to capture observed overall aggression or bullying, victimization, and defending using a scale (Veenstra and Huitsing 2021), or to directly nominate classmates in response to questions such as “Which classmates bully others?” (e.g., Huitsing and Veenstra 2012; Malamut et al. 2020; Obermann 2011; Raine et al. 2006). Each of the measurement approaches is valid but comes with its own set of challenges (Hunter, Noret, and Boyle 2021).

An important feature of the reputational nominations is that they rely on shared perceptions among students, as researchers typically calculate the average scores for each student and use various cut‐off classification schemes to assign peer roles such as aggressor, victim, or defender (Pouwels, Lansu, and Cillessen 2016). However, it has been documented that students in the same classroom may have different perspectives on who is involved in attacking others (Malamut et al. 2020), and those perspectives may be related to students' affective relationships of liking and disliking (Huitsing and Veenstra 2012; Huitsing et al. 2014). Thus, an important issue arises–it is possible that liking and disliking relationships shape students' perceptions of their classmates' aggressive acts toward others. On the one hand, friends of those participating in aggression may more likely to participate in the situations themselves (Huitsing and Veenstra 2012), and thus be more precise in knowing about who attacks who and who defends who. On the other hand, students may perceive those they like more favorably and those they dislike less favorably, hence, being more biased. This study investigates this issue and examines the moderating role of classroom bullying norms, as these norms determine which bullying roles are socially rewarded or sanctioned (Salmivalli and Voeten 2004). Thus, norms may modify students' motivation to support (dis)liked classmates in gaining social rewards or avoid social sanctions associated with various forms of involvement in aggression.

### 1.1 Social Support Theory

Social support theory offers a framework for understanding how students' affective relationships of liking and disliking may shape their perceptions of classmates' involvement in aggression. The theory suggests that friends provide mutual support, fostering a sense of moral commitment and obligation (Colvin, Cullen, and Ven 2002; Cullen 1994). This commitment often translates into loyalty towards friends (Brezina and Azimi 2018), which may influence how adolescents judge immoral behavior. Adolescents are likely to be more lenient when evaluating a friend, as they may share similar moral standards (Sijtsema et al. 2014) and prioritize loyalty over honesty to protect the friend (Shao et al. 2024). In classrooms, subgroups with distinct perspectives on aggression may form based on both liking and disliking (Huitsing and Veenstra 2012). Negative affective relationships can similarly bias judgments: adolescents may be less motivated to benefit, or may even exhibit negative biases against, classmates they dislike. Consequently, perceptions of a classmate's role as an aggressor, victim, or defender—and the willingness to admit these perceptions—may be shaped by the nature of the affective relationship with that classmate.

Similarly, social support may play a part in students' perceptions of involvement in aggression at a group level. It has been established that students form peer groups, with members within a peer group having strong and dense relationships with each other and weaker or less dense relationships with classmates outside the peer group (Espelage, Green, and Wasserman 2007; Hallinan and Smith 1989; Kindermann and Gest 2009). Both dyadic friendships and triadic friendship configurations are important sources of peer support (Wang et al. 2021). Therefore, if two or more peer groups are in conflict, students within the peer groups are more likely to perceive each other positively–for example, as defenders or victims–and those in the opposing peer group are more likely to be perceived as aggressors.

### 1.2 Evidence on the Role of Dyadic and Group Relationships

Previous evidence for the interaction between students' relationships and their perceptions of classmates' involvement in aggression is scarce. Using cross‐sectional data, Hanish et al. (2016) found that bullies and their friends were less likely to perceive bullies as such. Another investigation of cross‐sectional relational nominations showed that self‐reported bullies nominated classmates to be their victims more often when they disliked the classmates. A similar association was found from the perspective of self‐reported victims (Veenstra et al. 2007). Self‐reported victims were also more likely to nominate classmates as their defenders when they liked them (Oldenburg, Van Duijn, and Veenstra 2018; Rambaran et al. 2022). Corresponding effects for reputational peer nominations have not been examined.

Previous evidence for group‐level processes influencing students' perceptions of their classmates' involvement in aggression is missing, and we can draw only limited inferences from existing studies focusing on bullying group‐level processes in general. In network studies, group‐level processes have been operationalized as mixed triadic effects–that is, the tendency of students to form a certain type of tie toward another classmate, conditioned on the existence of other types of ties between the given student and a third student. It was found that when student A dislikes classmate B, and classmate B dislikes classmate C, there is a higher probability that student A will like classmate C (Huitsing et al. 2012). The authors pointed out that, at the structural level, the classroom structure of liking ties partially explained the structures of disliking and bullying ties (Huitsing et al. 2012). It was further found that there is a complex interdependency between disliking and defending, such that defending was more likely to occur between students who disliked the same classmates (Oldenburg, Van Duijn, and Veenstra 2018; Rambaran et al. 2022).

### 1.3 Evidence on the Role of Classroom Norms

Classroom‐level bullying norms, as the most immediate context shaping students' attitudes toward bullying (Pozzoli, Gini, and Vieno 2012; Salmivalli 2010), likely influence how students evaluate their classmates' behaviors during incidents of aggression. Relevant norms may include those approving aggression, such as classroom moral disengagement (Thornberg et al. 2021), as well as pro‐defending norms, such as peer pressure to support victimized peers (Pozzoli, Gini, and Vieno 2012). These norms establish the social costs and benefits associated with different types of involvement in aggression, including bullying (Salmivalli and Voeten 2004). Given that adolescents are attuned to the social rewards and punishments dictated by classroom norms (Salmivalli 2010), it is reasonable to expect that they may adjust their evaluations of classmates they like to align with the specific normative context of their classroom.

Only a few studies have touched upon this issue. Shin (2022) found that high bullying norms in classrooms are related to bullies being more likely to select other bullies as friends, suggesting that classroom‐level bullying norms might moderate students' willingness to ascribe certain bullying roles to their friends. Rambaran et al. (2022) found that being defended by someone whom one likes was less likely to happen in classrooms with high overall bullying occurrence, suggesting that pro‐bullying classroom norms make liking defenders less likely. Finally, Romera et al. (2019) found that both higher overall pro‐ and anti‐bullying classroom norms were linked to bullies and defenders being more popular, suggesting that strong bullying norms in classrooms make both bullies and defenders more likely to be liked.

### 1.4 Present Study

This study builds on the premise that investigating aggression in classrooms through students' reputational peer nominations, without considering their affective relationships of liking and disliking, risks introducing bias. As Vörös and Snijders (2017) emphasized, avoiding an ecological fallacy requires distinguishing between tie‐, individual‐, and group‐level processes, which necessitates modeling social relationships as a complex system.

To address this, the present study adopts a multiplex social network approach to explore how students' perceptions of involvement in physical aggression are shaped by their (dis)liking relationships. This approach enables the simultaneous examination of multiple types of relationships within the same network (Vörös and Snijders 2017), offering a critical framework to investigate how relationships influence perceptions of aggression, victimization, and defending roles in classroom settings. By dissecting nominations for involvement in aggression into specific ties between students, we explicitly model five interactive tie types: liking, disliking, and perceptions of aggressor, victim, and defender roles.

Unlike previous network‐based studies that sought to determine “who attacks whom” (e.g., Huitsing and Monks 2018; Veenstra and Huitsing 2021), this study does not aim to establish an objective truth about aggression among students. Instead, it focuses on understanding how dyad‐, group‐, and classroom‐level processes shape students' perceptions of aggression while accounting for the reciprocal effects of these perceptions on relationships. This approach avoids ecological fallacies by treating each perception as a unique tie rather than aggregating individual perceptions into student‐level attributes, as highlighted by Vörös and Snijders (2017).

Physical aggression, including bullying, is one of the most common and harmful forms of adolescent aggression (Jagasia et al. 2024; Tso et al. 2018), serves as a valid indicator of aggression in general, and is interconnect with classroom peer dynamics (Salmivalli et al. 1996). The decision to focus on physical aggression as a representative form of general aggression is supported by findings that various aggression forms often overlap, with students perceiving multiple forms simultaneously (Bradshaw, Waasdorp, and Johnson 2015).

This study's longitudinal design offers a significant advantage over prior cross‐sectional research (e.g., Hanish et al. 2016; Veenstra et al. 2007), allowing us to assess the directionality of interactions between students' relationships and their perceptions of peers' involvement in aggression. By addressing these dynamics, the study advances understanding of how relational processes influence perceptions of aggression within classroom contexts.

## 2 Hypotheses

Aligned with social support theory (Colvin, Cullen, and Ven 2002; Cullen 1994), we propose that dyad‐ and group‐level affective relationships influence students' perceptions of involvement in aggression, with this influence moderated by classroom‐level aggression norms.

The first set of hypotheses deals with the influence of dyadic affective relationships of liking and disliking on student's perceptions of peers' involvement in aggression. We expect liking a classmate to lead to a lower tendency to see them as an aggressor, and a higher tendency to see them as both a victim and a defender. We further expect that disliking a classmate leads to a higher tendency to see them as an aggressor and a lower tendency to see them as a defender. We do not have a specific expectation about the association between disliking a classmate and perceiving them as a victim. The first set of hypotheses is as follows:

Liking a classmate leads to a lower tendency to perceive them as an aggressor.

Liking a classmate leads to a higher tendency to perceive them as a victim.

Liking a classmate leads to a higher tendency to perceive them as a defender.

Disliking a classmate leads to a higher tendency to perceive them as an aggressor.

Disliking a classmate leads to a lower tendency to perceive them as a defender.

The second set of hypotheses address the influence of group processes on disliking and perceptions of involvement in aggression. We expect that liking a classmate who dislikes another classmate or perceives another classmate as having specific type of involvement in aggression influences one's tendency to dislike or perceive the other classmate the same way. Hence:

Liking a classmate who dislikes another classmate increases one's tendency to dislike the same classmate.

Liking a classmate who perceives another classmate as an aggressor increases one's tendency to perceive the same classmate as an aggressor.

Liking a classmate who perceives another classmate as a victim increases one's tendency to perceive the same classmate as a victim.

Liking a classmate who perceives another classmate as a defender increases one's tendency to perceive the same classmate as a defender.

The third set of hypotheses examines the moderating effect of classroom norms on the influence of affective relationships—liking and disliking—on students' tendencies to ascribe aggression, victimization, and defending roles to their classmates. We hypothesize that in classrooms characterized by high levels of moral disengagement or low levels of defending norms, students are more likely to ascribe aggressor roles to classmates they like and less likely to ascribe victimization and defending roles to those they like, compared to other classroom contexts. Conversely, we expect corresponding moderating effects in the opposite direction for the relationship between disliking and students' perceptions of involvement in aggression. Hence, the main expectations are as follows:

Higher classroom moral disengagement moderates the influence of dyadic and group liking and disliking on students' perceptions of involvement in aggression.

Higher classroom defending norms moderates the influence of dyadic and group liking and disliking on students' perceptions of involvement in aggression.

## 3 Methods

### 3.1 Participants

The study's sample involved 751 early adolescents (50.6% girls) from 39 seventh‐grade classrooms of 20 comprehensive elementary schools in Prague, the Czech Republic. The sample was age‐homogeneous (*M*_age_ at Time 1 = 12.9 years, SD = 0.4)–at the beginning of the study, most students were within the 12–13 years age range, while a small number were of different ages: one student was 11 years old, four were 14 years old, and one was 15 years old. The sample was mostly ethnically homogenous with 88.1% of the participants identifying as Czech. As data from some of the classrooms did not allow for an adequate convergence of the models (described in *5.6.1 SAOM* section), the effective sample size dropped to 27 classrooms comprising 632 students.

### 3.2 Procedure

We selected 28 comprehensive elementary schools in Prague at random and invited them to participate in our study, while 20 of the schools agreed to participate. Within each of the 20 participating schools, all seventh‐grade classrooms (ranging from 1 to 4 classrooms per school) were included in the sample. We collected data at two timepoints (T1 and T2) with an interval of 6 months (±2 weeks) within the same school year 2015/2016. All data were collected using pen‐and‐paper questionnaires administered in classrooms over two consecutive lessons. Trained research assistants facilitated the data collection.

This research was approved by the Ethics Committee of the Institute of Psychology of the Czech Academy of Sciences No. 656/Pha/17. All parents and legal guardians gave written informed consent to participation in the research. The study comprised students who had obtained written parental consent, were present at school during the study's administration, and willingly agreed to take part in it. No incentives or compensation were given to the participants. In each classroom, two trained research assistants were present during administration to ensure privacy and emotional safety. To maintain anonymity, we did not collect any personally identifiable data–all participants were assigned numerical codes for data storage. Following the project's conclusion, the participants received leaflets with basic information about bullying and effective responses to bullying (reporting the bullying to school personnel) and contact details for relevant hotlines.

### 3.3 Measures

#### 3.3.1 Affective Relationships

We used relational unlimited nominations to measure two distinct types of affective relationships among the students. Following the conceptualization and established measurements of positive and negative ties between students (Cillessen and Marks 2017), we used direct relational nominations to assess liking and disliking ties of students. The questions were constructed as “Who do you like most?” and “Who do you like least?”. We assigned values of 1 if a student nominated their classmate–indicating the presence of a tie–and a value of 0 if a student did not nominate their classmate–indicating the absence of the tie.

#### 3.3.2 Perceptions of Involvement in Physical Aggression

We used reputational peer nominations to assess how students perceived their classmates' involvement in situations of physical aggression. Perceptions of aggressors, victim, and defender roles were measured using single items adapted from a self‐report bullying, victimization, and defending scale developed by Pozzoli and Gini (2010). Participants were asked to rate each classmate based on how often they observed the classmate engaging in specific behaviors over the previous 2–3 months. The statements were:

- Aggressor: “He/she hits or pushes some classmates.”
- Victim: “Some classmates attack him/her hard or hit or push him/her.”
- Defender: “He/she defends classmates who are hit or attacked hard.”

Responses were recorded on a three‐point scale: never (1), sometimes (2), and often (3). For analysis, the response “never” was transformed into “0,” while “sometimes” and “often” were recoded as “1,” based on the assumption that any indication of the behavior from a student's perspective signifies their perception of the classmate's involvement in physical aggression.

This procedure resulted in five unweighted, directed adjacency matrices for each classroom, each representing a network of a specific type of tie: liking, disliking, and aggressors, victim, and defender nomination ties. Together, these matrices formed multiplex networks. The ties in each type of network were not mutually exclusive.

#### 3.3.3 Classroom Moral Disengagement (CMD)

Classroom collective moral disengagement scale (Gini, Pozzoli, and Bussey 2014) was used to assess classroom moral disengagement, that is, beliefs justifying bullying that are shared by classroom members. The originally Italian scale was validated in the Czech Republic by Kollerová, Soukup and Gini (2018a). The scale consists of 17 morally disengaged statements (e.g., “It is alright to beat someone who bad‐mouths your family.”) For each item, students rated “In your classroom, how many kids think that … [item].” Students marked their responses on the following 5‐point scale: “None,” “About a quarter (25%),” “About a half (50%),” “About three quarters (75%),” and “Everyone.” This variable was only measured at the second time point. To reduce the dimensionality of the measure, we fitted a unitary confirmatory factor analysis (CFA) model using the Weighted Least Squares–Mean and Variance Adjusted estimator, modeling the ordered nature of the items. We then computed the individual factor scores and aggregated them to the classroom level. The unidimensional factor structure had a mediocre fit to the data, with *χ* ^2^(119) = 462, *p* < 0.001; CFI = 0.91; TLI = 0.90; RMSEA = 0.071, 95% CI [0.064, 0.078].

#### 3.3.4 Classroom Defending Norms (CDN)

Classroom defending norms as shared perceived peer pressure to defend victimized classmates were assessed using a 3‐item measure (Kollerová et al. 2018b) introduced by the following instruction: “Classmates who are important to me think that I should …,”. The instruction was followed by three items related to defending classmates who have been victimized by physical, verbal, and relational aggression. Participants expressed their agreement with these statements on a 5‐point scale, ranging from “No” (1) to “Yes” (5). Data obtained at the second time point were used in the present study. Because there were only three items, we computed a unidimensional component score using the principal component analysis (PCA) instead of CFA and aggregated the individual scores at the classroom level. We do not report the model fit here because the model was just‐identified (loadings of all three items ranged from *λ* = 0.78 to 0.92).

#### 3.3.5 Student Covariates

To control the effects of gender on student relationships, we also collected data on students' gender.

### 3.4 Handling Missing Data

In the 27 classrooms included for the analysis, there were some missing ties of all types and no missing data on gender. At T1, we had on average 21.5% of missing liking, disliking, and aggressor, victim, and defender nomination ties. At T2, we had on average 26.8% of missing liking and disliking ties, and 26.1% of aggressor, victim, and defender nomination ties. The missing ties were treated as noninformative and dealt with a method described in detail in Huisman and Steglich (2008) as method four. In the first wave, missing entries within the adjacency matrices were assigned a value of 0, under the assumption of no ties, reflecting the common scenario of data sparsity where “no tie” is typically the modal value. For the second wave, the last observation carried forward approach was employed for all tie variables; tie value at T1 was used to impute the T2 value. The models were based on simulations carried out over all variables as if they were complete. However, to mitigate the impact of missing data treatment on parameter estimation, only nonmissing data were utilized for calculating the target statistics.

The attrition rates in the study were as follows: 443 students (70.1%) were present at both timepoints, 60 students (9.5%) were present only at T1, 31 students (4.9%) were present only at T2, and 98 students (15.5%) were not present at either timepoint but had incoming ties available. To address the potential issue of selective attrition, we compared basic demographic characteristics across four groups: students present at both timepoints, those present only at T1, those present only at T2, and those not present at any timepoint. The analysis revealed no statistically significant differences in age (ANOVA: *F*[2, 54] = 1.13, *p* = 0.33), gender (*χ*² = 0.57, *p* = 0.90), or ethnicity (*χ*² = 4.07, *p* = 0.13) between the groups.

### 3.5 Analytical Strategy

#### 3.5.1 Stochastic Actor Oriented Models

To test the first and the second set of hypotheses and capture the interacting mechanisms of affective relationships and the perceptions of peers' involvement in aggression, we used multiplex Stochastic Actor Oriented Models (SAOM; Snijders, Van de Bunt, and Steglich 2010; Snijders, Lomi, and Torló 2013) in *R* (R Core Team 2020) package *RSiena* (Ripley et al. 2011). Multiplex SAOMs are network models which can take a change on one type of tie between students as a dependent variable co‐evolving with other types of ties between the students while controlling for student characteristics and endogenous network mechanisms. In our case, we were interested in modeling changes on liking and disliking ties and changes on aggressor, victim, and defender nomination ties conditioned on each other. SAOMs are actor‐oriented meaning that tie change between the students is assumed to be the result of students' own choices about their outgoing ties. What changes students make is determined by the objective function, which contains the modeled effects capturing the hypothesized mechanisms, while how often they get to make these changes is determined by the rate function. Having two timepoints allowed us to assess the direction of the dependencies between the types of ties–for example, it allowed us to assess whether students tend to form liking towards those whom they consider defenders, or whether students tend to consider classmates as defenders if they had already formed liking towards them. SAOMs control for autoregression as they account for the influence of previous network states (in our case, T1) on tie formation in the following states (in our case, T2).

We obtained the parameter estimates and their standard errors (SEs) using the Method of Moments estimation technique. The tie change was operationalized by the evaluation function meaning that the parameter estimates were calculated on the presence of ties regardless of whether they were newly created or maintained from T1 to T2. In other words, we tested effects influencing simultaneously both the formation of new ties from T1 to T2 and the existence of ties at T1 that were maintained to T2. The resulting parameter estimates are in log odds ratios (log OR). Their positive values indicate that when the network configurations embodied by the given parameter are attractive for the students, they are more likely to create or maintain ties embedded within them, whereas negative values indicate the opposite.

Prior to the analysis, we conducted pilot convergence checks to screen out classrooms where the proportion of missing data was too large to provide an admissible solution for a given model. For all models included in the analysis, we also carried out post‐estimation convergence and goodness of fit (GoF) checks. Convergence issues denote situations when estimates from the model are expected to be biased due to algorithm not finding stable parameter estimates that adequately represent the observed social network dynamics. We used the criteria in Ripley et al. (2011) when assessing the convergence–the t‐ratios for convergence for all model terms are recommended to be < 0.10, and the overall maximum convergence ratio is recommended to be < 0.25. If convergence of a model was not achieved in a single estimation run, we engaged in a structured, automated series of estimations. First, in three runs, the algorithm attempted to improve convergence by using estimated parameters as starting values for the next estimation run. If not successful, three additional models were estimated, which increasingly used more iterations and smaller steps (reducing the scaling parameter). We provide the full description of the estimation procedures as Supplementary material–Appendix S.A. GoF, on the other hand, denotes the degree to which the model represents the observed real‐world network. GoF was assessed by simulating a distribution of 1000 networks from each converged model for each classroom and subsequently comparing this distribution to the observed data with respect to indegree and outdegree distributions, geodesic distances, and triad census. The models generally showed good fit on the liking networks with minor signs of misfit in terms of indegree and outdegree distributions, and signs of misfits on disliking networks in terms of triad censuses. The misfits on disliking networks in terms of triad censuses were probably the result of not having triadic effects on the disliking networks included. However, including them along with triadic effects on liking networks resulted in convergence issues, hence, we prioritized optimization for adequate convergence over goodness of fit on disliking ties.

Along with the Method of Moments estimation, we attempted Bayesian random‐coefficient multilevel estimation (Koskinen and Snijders 2023). Unfortunately, the Bayesian multilevel estimation is computationally demanding (see, e.g., Bravo et al. 2024), and we were unable to get any reasonable estimates within a reasonable timeframe on 64 cores.

#### 3.5.2 Meta‐Analysis and Meta‐Regression

After fitting the individual SAOMs, we transformed the original effect estimates into relative importances of effects, and we aggregated them using a meta‐analytical approach to get overall effect estimates over the classrooms. We used the Indlekofer‐Brandes measure of relative importance for effects (Indlekofer and Brandes 2013) to get comparable effect estimates from all classrooms. We then employed random‐effects meta‐analysis in *R* package *metafor* (Viechtbauer 2010), assuming that the observed estimates varied across the classrooms both because of real differences in the true effect sizes in each classroom and because of the sampling variability.

To test the third set of hypotheses and assess the moderating role of classroom bullying norms on the influence of dyadic and group liking and disliking on student's perceptions of peers' involvement in aggression, we conducted a series of meta‐regression analyzes moderating the results extracted from the meta‐analyzes by CMD and CDN, aiming to explain heterogeneity of variance in effect sizes between the studies. We conducted the meta‐regression separately for the three distinct model specifications as described in *5.7 Model specifications*. After estimating the effect in the baseline model, we cross‐validated the inference regarding the presence of the effect in more complex models. Once any effect was estimated using the more comprehensive models, we always selected primarily the model with a higher sample size. We provide the remaining effect estimates as Supplementary material–Appendix S.B.

Apart from using p‐values for error control, to evaluate the comparative evidence supporting the moderation effects, we employed Bayes factors (BF). Bayes factor offers insight into whether the data are more consistent with the null hypothesis (*H*_0_, effect absent, BF < 1), the alternative hypothesis (*H*_1_, effect present, BF > 1), or are inconclusive. Using a model‐selection/information‐criteria approach suggested by Wagenmakers (2007), we estimated BF through a BIC approximation, implicitly assuming a unit information prior. Alongside BF, we also computed the posterior probabilities for each parameter. These probabilities indicate the likelihood of the moderation parameter being different from zero, under the assumption of 1:1 prior odds for H_0_ and H_1_. We interpreted values of BF larger than 3 (or smaller than 1/3) as suggestive evidence and values larger than 10 (or smaller than 1/10) as strong evidence in favor of the respective hypothesis.

### 3.6 Model Specifications

**Figure 1:**
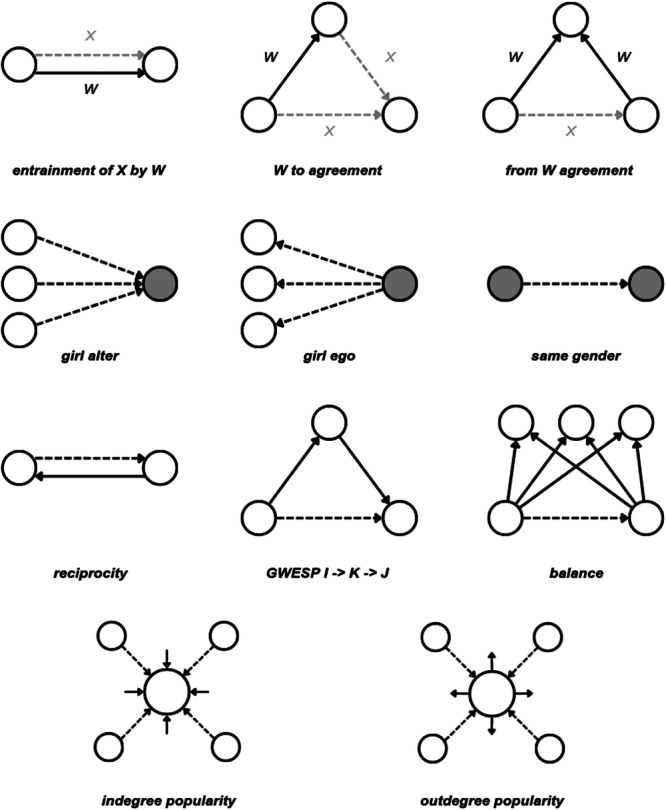
Diagrams of the included effects.

We fitted three distinct model specifications–a baseline model, a restricted model, and a full model. We fitted different specifications because the full model could not be fitted to most of the classrooms due to convergence issues. Hence, using a backward selection, we fitted the full model testing all our hypotheses, the restricted model testing our hypothesis without the victim perceptions, and the baseline model without both the victim perceptions and the disliking ties. We could fit the baseline model to 27 classrooms, the reduced model to 16 classrooms, and the full model to 8 classrooms. We included the following effects with Figure 1 showing the diagrams for the effects:

- *entrainment of X by W*–capturing the tendency of a student to form or maintain one type of tie with another student conditioned on the existence of another type of tie with the same student. With this term, we tested the first set of hypotheses relating to the dyad‐level interactions–the effects of affective relationships on perceptions of peers' involvement in aggression. We further used this term to control for the possible reversed direction of the relationship by including the effects of perceptions of peers' involvement in aggression on affective relationships.
- *W to agreement*–capturing the tendency of a student to form or maintain one type of tie with another student conditioned on the existence of the same type of tie towards the student from a third student with whom the first student has another type of tie. With this term, we tested the second set of hypotheses relating to the group‐level interactions–the effects of liking on shared disliking and perceptions of peers' involvement in aggression.
- *from W agreement*–capturing the tendency of a student to form or maintain one type of tie with another student conditioned on the existence of a shared different type of tie towards a third student from both students. With this term, we controlled for the possible reversed direction of the interactions included in the second set of hypotheses relating to the group‐level interactions–the effects of shared disliking and shared perceptions of peers' involvement in aggression on liking.
- *girl alter, girl ego, and same gender*–capturing the tendency of girls to receive or send more ties, and the tendency to form or maintain ties with students of same gender. With this term, we controlled the effects of gender on liking ties.
- *reciprocity*–controlling the tendency of students to reciprocate ties.
- *GWESP I ‐* > *K ‐* > *J* – controlling the tendency of students to form transitive liking ties.
- *balance*–controlling the structural equivalence with respect to outgoing ties, defined by the similarity between the outgoing ties of student A and the outgoing ties of the other students to whom student A is tied.
- *indegree and outdegree popularity*–controlling the tendency of students to receive or send disproportionately more liking ties if already receiving or sending many liking ties.
- *densities*–denoting the baseline tendency of students to form the individual types of ties.

## 4 Results

The results from the aggregate SAOM models show that at both dyad‐ and group‐level, student relationships influenced their perceptions of peers' involvement in aggression. Table 1 shows the overall model estimates. Supplementary material–Appendix S.C shows the network descriptive statistics, including network density, reciprocity, degree centralization indices, and Jaccard coefficients. Jaccard coefficients denoting the stability of ties were below 0.5 for all five tie types, suggesting that liking, disliking, as well as perceptions of peers' involvement in aggression we relatively unstable, with less than half of the ties persisting between T1 and T2.

**Table 1:** Overall model estimates.

	Baseline model (27 class.)			Reduced model (16 class.)			Full model (8 class.)		
	log OR	SE	*p*	log OR	SE	*p*	log OR	SE	*p*
***Effects of relationships on perceptions of peers' involvement in aggression***									
Entrainment of aggressor nomination by liking (H1a)	−0.006	0.077	0.936	0.033	0.133	0.804	0.178	0.145	0.219
Entrainment of victim nomination by liking (H1b)							0.089	0.105	0.399
Entrainment of defender nomination by liking (H1c)	0.348	0.062	< 0.001	0.306	0.087	< 0.001	0.387	0.102	< 0.001
Entrainment of aggressor nomination by disliking (H1d)				0.218	0.138	0.115	0.223	0.201	0.267
Entrainment of defender nomination by disliking (H1e)				−0.158	0.095	0.095	−0.106	0.135	0.431
***Effects of perceptions of peers whom one likes on one's own perceptions of peers; involvement in aggression***									
Liking to agreement on disliking (H2a)				0.398	0.069	< 0.001	0.287	0.085	0.001
Liking to agreement on aggressor nomination (H2b)				0.570	0.063	< 0.001	0.482	0.050	< 0.001
Liking to agreement on victim nomination (H2c)							0.375	0.057	< 0.001
Liking to agreement on defender nomination (H2d)				0.150	0.037	< 0.001	0.147	0.055	0.007
***control effects for perceptions of peers' involvement in aggression on relationships***									
Entrainment of liking by aggressor nomination	0.046	0.067	0.495	0.203	0.110	0.066	0.261	0.134	0.052
Entrainment of liking by victim nomination							−0.025	0.178	0.889
Entrainment of liking by defender nomination	0.235	0.086	0.006	0.337	0.144	0.020	0.398	0.172	0.021
Entrainment of disliking by aggressor nomination				0.218	0.075	0.004	0.279	0.101	0.006
Entrainment of disliking by defender nomination				−0.131	0.118	0.268	−0.102	0.172	0.555
***Control effects for shared perceptions of peers' involvement in aggression on relationships***									
Liking from disliking agreement				0.063	0.100	0.528	0.016	0.125	0.897
Liking from aggressor nomination agreement				−0.022	0.062	0.723	−0.009	0.070	0.901
Liking from victim nomination agreement							0.104	0.124	0.401
Liking from defender nomination agreement				−0.014	0.049	0.776	−0.043	0.060	0.470
***Control effects for gender***									
Girl alter on liking network	0.049	0.051	0.335	0.073	0.086	0.397	0.128	0.107	0.230
Girl ego on liking network	−0.022	0.064	0.725	0.007	0.083	0.933	0.007	0.081	0.929
Same gender on liking network	0.348	0.054	< 0.001	0.376	0.096	< 0.001	0.388	0.115	0.001
***Control structural effects***									
Reciprocity of liking	0.826	0.069	< 0.001	0.820	0.116	< 0.001	0.742	0.140	< 0.001
GWESP I ‐ > K ‐ > J on liking network	0.880	0.071	< 0.001	0.928	0.115	< 0.001	0.896	0.148	< 0.001
Balance on liking network	−0.003	0.029	0.913	−0.021	0.051	0.682	−0.014	0.063	0.821
Indegree popularity on liking network	−0.058	0.040	0.153	−0.127	0.063	0.043	−0.125	0.081	0.120
Outdegree popularity on liking network	−0.192	0.049	< 0.001	−0.227	0.080	0.004	−0.188	0.098	0.054
Reciprocity of disliking				0.371	0.070	< 0.001	0.428	0.115	<0.001
Reciprocity of aggressor nomination	0.142	0.064	0.026	0.252	0.066	< 0.001	0.294	0.091	0.001
Reciprocity of victim nomination							0.185	0.115	0.107
Reciprocity of defender nomination	−0.001	0.035	0.977	0.016	0.046	0.727	0.056	0.062	0.365
***Basic rate parameters and densities***									
Density on liking	−1.466	0.107	< 0.001	−1.414	0.174	< 0.001	−1.470	0.222	< 0.001
Density on disliking				−1.028	0.070	< 0.001	−1.042	0.105	< 0.001
Density on aggressor nomination	−0.595	0.062	< 0.001	−1.233	0.104	< 0.001	−1.219	0.148	< 0.001
Density on victim nomination							−1.145	0.089	< 0.001
Density on defender nomination	−0.412	0.059	< 0.001	−0.500	0.112	< 0.001	−0.547	0.144	< 0.001

At the dyad‐level, we found that liking a classmate increased the likelihood of nominating that classmate as a defender (H1c) with all three model specifications resulting in positive and significant effect estimates of *entrainment of defender nomination by liking*. On the other hand, we found no evidence that liking a classmate would increase the likelihood of nominating that classmate as an aggressor (H1a) or a victim (H1b) with *entrainment of aggressor nomination by liking* and *entrainment of victim nomination by liking* being non‐significant. We further found that the liking‐defender nomination relationship is bidirectional as nominating a classmate as a defender also increases the likelihood of liking that classmate with all three model specifications resulting in positive and significant effect estimates of *entrainment of liking by defender nomination*. We did not find evidence for the effects of disliking on either nominating that classmate as an aggressor (H1d) or a defender (H1e).

At the group‐level, we found evidence for the influence of liked classmates on one's perceptions of peers' involvement in aggression, while we found no evidence running the other direction for the influence of having shared perceptions of peers' involvement in aggression on formation of new liking ties. All observed effects of perceptions of peers whom one likes on one's own perceptions of peers' involvement in aggression were significant and positive. *Liking to agreement on disliking* effect suggests that liking a classmate who dislikes another classmate increased one's likelihood of also disliking that another classmate (H2a). *Liking to agreement on aggressor, victim*, and *defender nomination* effects suggest that liking a classmate who nominates another classmate as an aggressor (H2b), a victim (H2c), or a defender (H2d) increased one's likelihood of also nominating that another classmate as an aggressor, a victim, or a defender. Controlling for the other direction of the interactions, we found no evidence for the effects of shared aggressor, victim, and defender nominations on relationships, with all effects being non‐significant.

Control effects for gender and structure confirmed the influence of gender, reciprocity, transitivity, and outdegree popularity on formation of liking ties. Positive *same gender* effect suggests that students tended to like same‐gender classmates. *Gender alter* and *ego on liking network* effects are not significant suggesting that neither girls nor boys tended to like more classmates or be liked by more classmates. *Reciprocity of liking* and *GWESP I ‐* > *K ‐* > *J on liking network* effects are strong, positive, and significant suggesting that formation of liking ties was mostly influenced by the tendency of students to like classmates who also like them and to like classmates who were liked by others whom the student liked as well. Negative and significant *outdegree popularity on liking* effect suggests that there was a general tendency of students to like those classmates who did not like many others. We further found positive and significant effects of *reciprocity of disliking* and *reciprocity of aggressor nomination* suggesting that students tended to dislike those classmates who disliked them and nominate those classmates as aggressors who also perceived them as aggressors.

Results from the meta‐regression show that high CMD reduced the influence of defender nominations of classmates whom one liked on one's own perceptions, possibly increased the influence of liking on nominating classmate as an aggressor, and that high CDN possibly reduced the influence of affective relationships on aggressor nominations. Table 2 shows the meta‐regression results.

**Table 2:** Meta‐regression results.

	Baseline model (27 class.)			Reduced model (16 class.)			Full model (8 class.)		
	log OR	SE	BF_10_	log OR	SE	BF_10_	log OR	SE	BF_10_
***Moderation effects of classroom moral disengagement***									
Entrainment of aggressor nomination by liking	0.110	0.194	0.225	0.887	0.363	2.835	1.118	0.518	3.029
Entrainment of victim nomination by liking							0.751	0.509	1.120
Entrainment of defender nomination by liking	−0.160	0.181	0.284	0.123	0.302	0.301	−0.096	0.429	0.387
Entrainment of aggressor nomination by disliking				−0.120	0.497	0.285	−0.433	0.859	0.428
Entrainment of defender nomination by disliking				−0.158	0.353	0.306	−0.641	0.522	0.803
Liking to agreement on disliking				−0.215	0.216	0.445	0.041	0.333	0.381
Liking to agreement on aggressor nomination				−0.137	0.208	0.331	−0.465	0.240	2.483
Liking to agreement on victim nomination							−0.084	0.232	0.404
Liking to agreement on defender nomination				−0.346	0.123	13.423	−0.586	0.206	17.084
***Moderation effects of classroom defending norms***									
Entrainment of aggressor nomination by liking	−0.120	0.244	0.217	−0.647	0.483	0.658	−8.019	3.278	5.357
Entrainment of victim nomination by liking							−5.211	2.611	2.768
Entrainment of defender nomination by liking	0.459	0.181	4.137	0.401	0.284	0.689	−2.867	2.299	0.761
Entrainment of aggressor nomination by disliking				−0.260	0.522	0.314	−8.227	3.115	4.801
Entrainment of defender nomination by disliking				−0.724	0.302	4.861	0.881	2.378	0.405
Liking to agreement on disliking				−0.095	0.260	0.296	−2.166	1.449	1.034
Liking to agreement on aggressor nomination				−0.089	0.269	0.293	1.999	1.436	0.996
Liking to agreement on victim nomination							0.831	1.110	0.500
Liking to agreement on defender nomination				−0.046	0.154	0.290	0.793	1.371	0.446

Starting with the moderating role of CMD (H3a), we found a significant moderation effect on *liking to agreement on defender nomination*. It is negative and significant in both reduced (BF_10_ = 13.423) and full model (BF_10_ = 17.048), we therefore interpret this as a strong indication that high CMD reduced the influence of nominating peers whom one liked as defenders. We also found some indication that a higher CMD was related to a higher tendency of students to nominate peers they like as aggressors with *entrainment of aggressor nomination by liking* effect significant in the full model (BF_10_ = 3.029), but only indicative in the reduced model (BF_10_ = 2.835) and non‐significant in the baseline model (BF_10_ = 0.225). We found no evidence that CMD would moderate any other dyad‐ or group‐level processes.

The evidence for the moderating role of CDN (H3b) is mixed. In the full model, we found a negative and significant moderating effect of CDN on *entrainment of aggressor nomination by liking* and *entrainment of aggressor nomination by disliking* suggesting that in classrooms with high level of defending norms, the effects of both liking (BF_10_ = 5.357) and disliking (BF_10_ = 4.801) a classmate decreased the likelihood of nominating that classmate as an aggressor. However, these results were mainly driven by a single outlier classroom in the full model comprising only eight classrooms. In the reduced and baseline models, the two effects have the same negative direction, but they are not significant. We therefore interpret this only as suggestive evidence. We further found a positive and significant moderating effect of CDN on *entrainment of defender nomination by liking* in the baseline model (BF_10_ = 4.137) suggesting that higher pro‐defending classroom norms increased the tendency of students to nominate classmates as defenders if they liked them. However, the moderating effect is not significant in the reduced and full models. We therefore again interpret this only as suggestive evidence. Finally, we found contradictory evidence for the effect of CDN on *entrainment of defender nomination by disliking* effect; in the reduced model, there is an indication that higher pro‐defending classroom norms were linked to lower tendency of students to nominate classmates as defenders if they disliked that classmate (BF_10_ = 4.861), however, in the full model, this effect was not significant and was in the opposite direction (BF_10_ = 0.405). We found no evidence that CDN would moderate any other dyad‐level or group‐level processes.

## 5 Discussion

The present study investigated an understudied problem of the influence of affective relationships of (dis)liking between students and their perceptions of peers' involvement in physical aggression (aggressor, victim, and defender nominations). The investigation was based on social support theory (Colvin, Cullen, and Ven 2002; Cullen 1994), assuming that when students evaluate potential aggressor, victim, and defender role of a classmate, they may be influenced by their own and their friends' liking and disliking of that classmate, and that these associations could further be shaped by classroom norms that define social rewards and costs for various behaviors (Salmivalli and Voeten 2004). Previous research was missing despite the widespread use of peer nominations to assess students' involvement in aggressive behavior. Our study has shown that the perceptions of students may be biased by their affective relationships and moderated by classroom moral disengagement and classroom defending norms. Considering the role of affective relationships in nominations for involvement in aggression might therefore increase the accuracy of the peer‐reported bullying screening methods. On a dyadic level, liking a classmate increased the likelihood of perceiving that classmate as a defender (see first set of hypotheses). On a group level, perceptions of peers' involvement in aggression adopted by classmates whom one liked influenced one's own subsequent perceptions of this classmate including whether the classmate attacked others, was victimized, or defended others (see second set of hypotheses). At a classroom level, high classroom moral disengagement decreased the influence effect of defender nominations adopted by classmates whom one likes, and classroom defending norms provided inconclusive results (see third set of hypotheses). In sum, the findings document that perceptions of aggression, victimization, and defending are intertwined with liking among classmates and that classroom normative context enters these complex associations.

The question is, why, on the dyadic level, we did not find influence of affective relationships on aggressor nominations. One plausible explanation could be that physical aggression, being a more overt and often observable form of aggression (Zhang, Liu, and Zhang 2020), may not require a personal liking or disliking relationship to be recognized. It might be relatively easy for students to identify physically aggressive behaviors objectively, without their personal relationships with the involved individuals significantly influencing their perceptions. Many students directly use aggression, including physical aggression, to make themselves more visible and popular, which makes their behavior more easily recognized by their classmates (Salmivalli 2014). Unlike defending behaviors, which may be more subtle or ambiguous, and thus more likely to be influenced by personal relationships, acting aggressively could be blatant enough to be recognized regardless of the observer's affective ties.

Another question is why we found a strong and significant effect of reciprocated aggressor nominations. Our finding points to the possibility of bidirectional antagonism, where individuals may alternate in roles as aggressors and victims within a dyadic relationship or a group setting. This finding aligns with previously documented evidence of reciprocity in relational aggression nominations, which was interpreted as a sign that aggression may sometimes be bidirectional (Huitsing and Monks 2018; Kisfalusi, Pál, and Boda 2020). This complexity may challenge the common narrative of a unidirectional aggressor‐victim relationships in classrooms and calls for a deeper exploration of the multi‐faceted interactions that constitute aggressive behaviors. The bully‐victim double roles are prevalent, and a portion of this phenomenon might be anchored in reality, if the victim gains confidence for a revenge, original bullies might perceive this act as bullying and not as a self‐defense or equating mutual injustices (Sarıçam and Çetinkaya 2018). Also, more research is needed to compare levels of reciprocity across different aggression forms, as differences in tendency to retaliate and related reciprocity could be expected in various forms of aggression (Stubbs‐Richardson and May 2021).

Our group‐level findings support the view that students form subgroups with different perspectives on what is happening in the classroom. The study by Huitsing and Veenstra (2012) previously revealed that the common research practice of assigning participant roles to students based on averaged received reputational nominations may be limited, as students may form heterogeneous subgroups that may act aggressively toward some classmates and defend others. Specifically, it showed that some students, when nominated for defending in response to a reputational question, defended aggressors rather than victims (Huitsing and Veenstra 2012). Building upon this knowledge, our results reveal that these subgroups may even have differing perspectives on who adopts the roles of aggressor, victim, or defender within the classroom dynamics.

Our findings suggest that shared liking between two students may lead to shared perceptions of peers' involvement in aggression. However, the reverse does not hold true. This finding offers some evidence supporting social support theory at the group level (Colvin, Cullen, and Ven 2002; Cullen 1994). Assuming that students who like each other are likely part of the same peer group, the development of shared perceptions over time might reflect a group‐level dynamic, where conflicts arise between opposing groups. In such cases, perceptions of aggression could serve to support in‐group peers rather than out‐group peers, emphasizing group loyalty over individual relationships. However, we were unable to determine the extent to which the influence of liking on aggressor, victim, and defender nominations resulted from actual aggression dynamics or perception bias. Previous research (Huitsing et al. 2014; Huitsing and Monks 2018) has shown that aggression and defending behaviors are not random—for instance, victims of the same aggressors often defend each other. It is plausible that perceiving another student as a defender might stem from both liking that student and being defended by them. With our reliance solely on peer‐report questionnaires, we could not disentangle these mechanisms. Another plausible explanation is that students have more accurate perceptions of the defending behaviors of peers they like, as they are more familiar with these peers' actions, while underestimating such behaviors in peers they do not like.

Our study partially supports the idea that classroom norms moderate the interactions between liking and nominations for aggressor and defender roles. Specifically, we found evidence that classroom moral disengagement diminishes the influence of liking on defender nominations. In morally disengaged classrooms, where defending is associated with higher social costs (Thornberg et al. 2021), students may be less likely to ascribe the defender role to a liked classmate to help them avoid the social repercussions of defending in such an unfavorable context. This finding is consistent with the results of Rambaran et al. (2022). However, we did not confirm the moderating effect of pro‐bullying classroom norms reported in prior studies. Unlike Shin (2022), who found that aggressors are more likely to select other aggressors as friends in pro‐bullying classrooms, and Romera et al. (2019), who reported greater willingness to like aggressors in such contexts, our findings do not support these patterns. Additionally, we found tentative evidence that in classrooms with strong pro‐defending norms, students may be more likely to ascribe the defender role to those they like, possibly because defending is a valued trait in these environments (Pozzoli, Gini, and Vieno 2012).

### 5.1 Limitations

Our study has several limitations related to the sample and the instruments used. First, the initial sample of 39 classrooms was reduced to 27 classrooms due to model convergence issues, and some hypotheses were tested on even smaller subsamples. This reduction may have limited our ability to detect smaller yet theoretically relevant effects due to insufficient statistical power. Computing constraints prevented the use of a Bayesian multilevel approach, which might have accommodated a larger sample size. Additionally, we focused on three commonly studied roles in peer aggression: aggressor, victim, and defender. However, students can occupy other roles, such as reinforcer, assistant, or outsider, as described by Salmivalli et al. (1996). Furthermore, we assessed only physical aggression, which may interact differently with students' affective relationships compared to other forms of aggression in schools. Finally, our findings might be influenced by data not missing at random. While we found no significant differences in basic demographic characteristics among students with varying levels of attrition, unmeasured factors may have contributed to the missing data, potentially introducing bias.

### 5.2 Implications

Our research offers valuable implications for future studies on aggression and multiplex networks. We demonstrate that incorporating affective relationships of liking between students is essential in relational research on aggression. Failing to account for these relationships risks biasing findings, as students' perceptions of classmates as aggressors, victims, or defenders may be heavily influenced by liking. Accounting for this perception bias could enable more targeted interventions, distinguishing, for example, peer group conflicts from genuine incidents of aggression. Future studies could enhance validity by utilizing self‐reported data on involvement in aggression to cross‐validate peer nominations. Researchers might also replicate our study using relational or verbal aggression forms and conduct sensitivity analyzes with physical aggression data. Additionally, we support Vörös and Snijders (2017) argument that multiplex network models help avoid the ecological fallacy by disentangling tie‐, individual‐, and group‐level processes. The multiplex approach proved critical for examining interactions among different types of ties without conflating ties with individual attributes. We showed that multiplex stochastic actor‐oriented models (SAOMs) are suitable for analyzing small networks with complex tie specifications. However, the complexity of models involving three, four, or five interacting tie types often led to convergence challenges. Future research could benefit from developing multiplex network models that are more robust to convergence issues, ensuring their applicability in broader contexts.

## Conflicts of Interest

The authors declare no conflicts of interest.

## Data Availability

The data, analytical code, and the supplementary materials are available at OSF at https://osf.io/7edcp/.
